# ﻿Southbound – the southernmost record of *Tylototriton* (Amphibia, Caudata, Salamandridae) from the Central Highlands of Vietnam represents a new species

**DOI:** 10.3897/zookeys.1168.96091

**Published:** 2023-07-03

**Authors:** Trung My Phung, Cuong The Pham, Truong Quang Nguyen, Hoa Thi Ninh, Huy Quoc Nguyen, Marta Bernardes, Son Thanh Le, Thomas Ziegler, Tao Thien Nguyen

**Affiliations:** 1 Dong Khoi 9A, Tam Hiep, Bien Hoa, Dong Nai Province, Vietnam Unaffiliated Bien Hoa Vietnam; 2 Institute of Ecology and Biological Resources, Vietnam Academy of Science and Technology, 18 Hoang Quoc Viet Road, Hanoi 10072, Vietnam Institute of Ecology and Biological Resources, Vietnam Academy of Science and Technology Hanoi Vietnam; 3 Graduate University of Science and Technology, Vietnam Academy of Science and Technology, 18 Hoang Quoc Viet Road, Cau Giay, Hanoi 10072, Vietnam Graduate University of Science and Technology Hanoi Vietnam; 4 Institute of Genome Research, Vietnam Academy of Science and Technology, 18 Hoang Quoc Viet Road, Hanoi 10072, Vietnam Institute of Genome Research, Vietnam Academy of Science and Technology Hanoi Vietnam; 5 Vietnam National Museum of Nature, Vietnam Academy of Science and Technology, 18 Hoang Quoc Viet Road, Hanoi 10072, Vietnam Vietnam National Museum of Nature, Vietnam Academy of Science and Technology Hanoi Vietnam; 6 Cologne Zoo, Riehler Str. 173, D–50735 Cologne, Germany Cologne Zoo Cologne Germany; 7 Institute of Zoology, University of Cologne, Zülpicher Str. 47b, D–50674 Cologne, Germany University of Cologne Cologne Germany; 8 National Institute of Medicinal Materials, 43 Đinh Tien Hoang, Ben Nghe, Ho Chi Minh city, Vietnam National Institute of Medicinal Materials Ho Chi Minh City Vietnam

**Keywords:** Crocodile newt, ND2 gene, Ngoc Linh Mountain, Salamandridae, taxonomy, *Tylototritonngoclinhensis* sp. nov.

## Abstract

A new species of the genus *Tylototriton* is described from Ngoc Linh Mountain, Kon Tum Province, in the Central Highlands of Vietnam based on integrative taxonomy, namely by combining molecular and morphological evidence. *Tylototritonngoclinhensis***sp. nov.** differs from all other congeners based on morphological data, allopatric distribution, and molecular divergence. In terms of genetic divergence, *Tylototritonngoclinhensis***sp. nov.** distinctly differs from the sister species *T.panhai* (6.77%) and from *T.ngarsuensis* (12.36%) based on the mitochondrial NADH dehydrogenase subunit 2 (ND2) gene. *Tylototritonngoclinhensis***sp. nov.** is a moderate sized and robust salamander species with large cephalic edges, parotoids, and vertebral ridge orange in coloration. The new taxon differs from its congeners by a combination of the following morphological characteristics: size medium (SVL 60.8–66.5 mm, TL 57.6–61.8 mm in males, and SVL 72.5–75.6 mm, TL 62.9–67.9 mm in females); head longer than wide; parotoids very prominent and enlarged, projecting backwards; tail length shorter than snout-vent length; vertebral ridge large, high and glandular in appearance; 14 large and distinct dorsolateral glandular warts; gular fold present; tips of fore and hind limbs overlapping when adpressed along the body; tips of fingers reaching between eye and nostril when foreleg is laid forward; dorsal surface and lateral sides of the head, upper and lower lips, dorsolateral glandular warts, vertebral ridge, the peripheral area of the cloaca and the ventral edge of the tail orange in coloration; the presence of a distinct black line extending from the posterior end of the eye towards the shoulder. *Tylototritonngoclinhensis***sp. nov.** is restricted to evergreen montane forests near water bodies on Ngoc Linh Mountain. We suggest that the new species should be classified as Endangered (EN) in the IUCN Red List. This new important discovery represents the eighth *Tylototriton* taxon described from Vietnam, and at the same time constitutes the southernmost distributional record for the whole genus in Asia.

## ﻿Introduction

The salamandrid genus *Tylototriton* Anderson, 1871, commonly known as crocodile newts, currently contains 38 species inhabiting montane forest areas throughout the Asian monsoon climate zone and is distributed across Asia, from eastern Himalayas, eastern Nepal, northern India, Bhutan, Myanmar, central to southern China (including Hainan Island), and southwards through Laos, Thailand, and Vietnam (Dufresnes and Hernandez 2022; Li et al. 2022; Wang et al. 2022; Frost 2023).

Remarkably, 15 new species have been described in the past five years (Hernandez 2016; Dufresnes and Hernandez 2022; Frost 2023). The genus was subdivided into three subgenera, *Tylototriton*, *Yaotriton*, and *Liangshantriton* (e.g., Dubois and Raffaëlli 2009; Nishikawa et al. 2013a; Bernardes et al. 2020; Pomchote et al. 2021) and includes several, as yet, unnamed taxa, which contain cryptic species that are morphologically difficult to distinguish, especially in their southern range of distribution (Hernandez 2016; Pomchote et al. 2021; Poyarkov et al. 2021b). Most of the new discoveries derived from the splitting of widely distributed *Tylototriton* taxa through the efforts of integrative taxonomy, namely the combination of morphological and phylogenetic analyses.

In Vietnam, six species and seven taxa are currently known, *Tylototritonanguliceps* Le, Nguyen, Nishikawa, Nguyen, Pham, Matsui, Bernardes & Nguyen, 2015; *T.pasmansipasmansi* Bernardes, Le, Nguyen, Pham, Pham, Nguyen & Ziegler, 2020; *T.pasmansiobsti* Bernardes, Le, Nguyen, Pham, Pham, Nguyen & Ziegler, 2020; *T.sparreboomi* Bernardes, Le, Nguyen, Pham, Pham, Nguyen & Ziegler, 2020; *T.thaiorum* Poyarkov, Nguyen & Arkhipov, 2021; *T.vietnamensis* Böhme, Schöttler, Nguyen & Köhler, 2005; and *T.ziegleri* Nishikawa, Matsui & Nguyen, 2013 (Bernardes et al. 2017a; 2017b; Poyarkov et al. 2021b; Raffaëlli 2022; Frost 2023). All afore mentioned species are known from northern Vietnam, from Ha Giang Province southwards to Nghe An Province.

During recent fieldwork in May 2022 a new *Tylototriton* population was discovered in Ngoc Linh Mountain, Kon Tum Province, Central Vietnam resembling the *T.panhai* phenotype I from Phu Luang Wildlife Sanctuary and Phu Ruea National Park, Loei Province, northeastern Thailand (see Hernandez 2016; Hernandez and Pomchote 2020). However, morphological comparisons and molecular phylogenetic analyses revealed this southernmost *Tylototriton* population to be distinct from all related species of the genus. We herein describe the newly discovered *Tylototriton* population from the Central Highlands of Vietnam as a new species.

## ﻿Materials and methods

### ﻿Sampling

A field survey was conducted in Ngoc Linh Nature Reserve, Kon Tum Province of the Central Highlands, Vietnam, on 22^nd^ of May 2022. Crocodile newts were found on the forest floor between 9:00 and 16:00. After having been photographed in life, six specimens were anaesthetized and euthanized in a closed vessel with a piece of cotton wool containing ethyl acetate (Simmons 2002), fixed in 80% ethanol for five hours, and then later transferred to 70% ethanol for permanent storage. Muscle tissue from the central upper part of the tail was taken for tissue samples, which were preserved separately in 70% ethanol prior to fixation. Voucher specimens referred to in this paper were deposited in the collections of the Institute of Ecology and Biological Resources (IEBR), Hanoi, Vietnam.

### ﻿Molecular analyses

DNA from tissue samples of the preserved specimens were extracted using the Dneasy blood and tissue kit, Qiagen (California, USA). A fragment of a mitochondrial gene, the NADH dehydrogenase subunit 2 (ND2), was amplified by PCR master mix (Fermentas, Burlington, ON, Canada) using the primer pair, Sal_Nd2_F1 (5’- AAGCTTTTGGGCCCATACC-3’), Sal_Nd2_R1 (5’-GTTATAAATATGGAKLARGTTA-3’) (Nishikawa et al. 2013b).

For the phylogenetic analyses, 53 sequences of species of the genus *Tylototriton* were used in combination with a sequence of *Pleurodeleswaltl* and *Echinotritonchinhaiensis* as outgroups according to Li et al. (2022) (Table 1).

**Table 1.** Samples of the *Tylototriton* species and other species used for DNA analyses in this study.

No.	Scientific name	Voucher number	Locality	GenBank number	Reference
1.	*Tylototritonngoclinhensis* sp. nov.	IEBR A.5131	Kon Tum Prov., Vietnam	LC575223	This study
2.	*Tylototritonngoclinhensis* sp. nov.	IEBR A.5130	Kon Tum Prov., Vietnam	LC575221	This study
3.	*Tylototritonngoclinhensis* sp. nov.	IEBR A.5133	Kon Tum Prov., Vietnam	LC575222	This study
4.	*T.anguliceps*	NUOL 00420	Viengphoukha, Luang Namtha, Laos	KT304301	Phimmachak et al. (2015)
5.	*T.anhuiensis*	CIB 08042905-3	Yuexi Co. Anhui Prov., China	KY800854	Wang et al. (2018)
6.	*T.anhuiensis*	CIB 08042905-4	Yuexi Co. Anhui Prov., China	KY800855	Wang et al. (2018)
7.	*T.asperrimus*	CIB GX20080714	Jinxiu Co., Guangxi Prov., China	KY800819	Wang et al. (2018)
8.	*T.broadoridgus*	CIB 200084	Sangzhi Co., Hunan Prov., China	KY800837	Wang et al. (2018)
9.	*T.dabienicus*	HNNU1004-024	Shangcheng Co., Henan Prov., China	KC147812	Nishikawa et al. (2014)
10.	*T.dabienicus*	HNNU 1004-015	Shangcheng Co., Henan Prov., China	KC147811	Nishikawa et al. (2014)
11.	*T.dabienicus*	HNNU 1004-026	Shangcheng Co., Henan Prov., China	KY800869	Wang et al. (2018)
12.	*T.daloushanensis*	GZNU 20060626002	Suiyang Co., Guizhou Prov., China	JF825872	Li et al. (2022)
13.	*T.daloushanensis*	GZNU 20060626001	Suiyang Co., Guizhou Prov., China	FJ415600	Wang and Gu (2008)
14.	*T.hainanensis*	CIB 20081048	Mt. Diaoluo, Hainan Prov., China	KC147817	Nishikawa et al. (2013b)
15.	*T.himalayanus*	CIB 201406246	Mai Pokhari, Illam, Mechi, Nepal	KT765173	Khatiwada et al. (2015)
16.	*T.kweichowensis*	CIB Wg20080818014	Bijie City, Guizhou Prov., China	KY800823	Wang et al. (2018)
17.	*T.liuyangensis*	CIB 110601F06	Liuyang City, Hunan Prov., China	KY800875	Wang et al. (2018)
18.	*T.maolanensis*	CIB ML20180427003	Libo Co., Guizhou Prov., China	MK820701	Li et al. (2020)
19.	*T.maolanensis*	CIB ML20180427004	Libo Co., Guizhou Prov., China	MK820702	Li et al. (2020)
20.	*T.maolanensis*	GZNU 200706050101	Leishan Co., Guizhou Prov., China	FJ415596	Li et al. (2020)
21.	*T.maolanensis*	GZNU 200706050102	Leishan Co., Guizhou Prov., China	JF825868	Li et al. (2020)
22.	*T.ngarsuensis*	LSUHC13762	Shan State, Myanmar	MH836585	Grismer et al. (2018)
23.	*T.notialis*	FMNH: HERP:271120	Boualapha, Khammouan, Laos	HM462061	Stuart et al. (2010)
24.	*T.panhai*	NUOL 00424	Botene, Xaignabouli, Laos	KT304309	Phimmachak et al. (2015)
25.	*T.panhai*	NUOL 00425	Botene, Xaignabouli, Laos	KT304311	Phimmachak et al. (2015)
26.	*T.panhai*	NUOL 00421	Botene, Xaignabouli, Laos	KT304310	Phimmachak et al. (2015)
27.	*T.pasmansi*	IEBR 4466 (Holotype)	Da Bac, Hoa Binh Prov., Vietnam	MT210166	Bernardes et al. (2020)
28.	*T.pasmansi*	IEBR:4467	Da Bac, Hoa Binh Prov., Vietnam	MT210167	Bernardes et al. (2020)
29.	*T.podichthys*	NCSM 77725	Phoukhoun, Luang Phabang, Laos	KT304295	Phimmachak et al. (2015)
30.	*T.pseudoverrucosus*	CIB WCG2012003	Ningnan Co., Liangshanyizu State, Sichuan Prov., China	KY800861	Wang et al. (2018)
31.	*T.pulcherrimus*	KUHE:46406	Pet Trade	KY800880	Wang et al. (2018)
32.	*T.shanjing*	CIB 980004	Baoshan City, Yunnan Prov., China	KY800831	Wang et al. (2018)
33.	*T.shanorum*	KUHE 42348	Shan State, Myanmar	AB769544	Nishikawa et al. (2013b)
34.	*T.sini*	SYS a008354	Mt Yunkai, Guangdong Prov., China	OK539836	Lyu et al. (2021)
35.	*T.sparreboomi*	IEBR 4477	Sin Ho, Lai Chau Prov., Vietnam	MT210163	Bernardes et al. (2020)
36.	*T.taliangensis*	CIB GG200110183	Shimian Co., Yan’an City, Sichuan Prov., China	KC147819	Yang et al. (2014)
37.	*T.thaiorum*	ZMMU A-7577	Pu Hoat NR, Nghe An Prov., Vietnam	MW883478	Poyarkov et al. (2021a)
38.	*T.tongziensis*	CIB WB2020081511	Tongzi Co., Guizhou Prov., China	OK349411	Li et al. (2022)
39.	*T.tongziensis*	CIB TZ20160714002	Tongzi Co., Guizhou Prov., China	OK349413	Li et al. (2022)
40.	*T.tongziensis*	TZ20160714010	Tongzi Co., Guizhou Prov., China	OK349414	v
41.	*T.tongziensis*	CIB WB2020202	Tongzi Co., Guizhou Prov., China	OK349415	Li et al. (2022)
42.	*T.uyenoi*	KUHE:19147	Doi Suthep, Chiang Mai Prov., Thailand	AB830733	Nishikawa et al. (2013a)
43.	*T.verrucosus*	CIB-TSHS1	Longchuan Co., Dehong State, Yunnan Prov., China	KY800847	Wang et al. (2018)
44.	*T.wenxianensis*	CIB 2010123101	Pingwu Co., Gansu Prov., China	KY800867	Wang et al. (2018)
45.	*T.wenxianensis*	CIB 2010123102	Pingwu Co., Gansu Prov., China	KY800868	Wang et al. (2018)
46.	*T.yangi*	KUHE:42282	Pet Trade	KY800887	Nishikawa et al. (2014)
47.	*T.ziegleri*	VNMN 3390	Quan Ba, Ha Giang Prov., Vietnam	KY800889	Wang et al. (2018)
48.	*T.liuyangensis*	CSUFT20100108	Liuyang City, Hunan Prov., China	KJ205598	Yang et al. (2014)
49.	*T.phukhaensis*	CUMZ-A-7717	DPKNP, Nan Prov., Thailand	MN912573	Pomchote et al. (2020)
50.	*T.sparreboomi*	IEBR 4477	Sin Ho, Lai Chau Prov., Vietnam	MT210163	Bernardes et al. (2020)
51.	*T.vietnamensis*	IEBR A.2014.44	Mau Son, Loc Binh, Lang Son Prov., Vietnam	KX609962	Bernardes et al. (2017a)
52.	*T.vietnamensis*	IEBR A.2014.43	Hoanh Bo, Quang Ninh Prov., Vietnam	KX609961	Bernardes et al. (2017a)
53.	*T.vietnamensis*	IEBR A.0701	Mau Son, Lang Son Prov., Vietnam	KY800873	Wang et al. (2018)
54.	*Echinotritonchinhaiensis*	CIB ZHJY2	Zhenhai Co., Zhejiang Prov., China	KY800892	Wang et al. (2018)
55.	*Pleurodeleswaltl*	-	Cadiz, Andalusia, Spain	EU880330	Zhang et al. (2008)

CHROMAS PRO software (Technelysium Pty Ltd., Tewantin, Australia) was used to edit the sequences, which were aligned using MAFFT v. 7 (Katoh and Standley 2013) with default settings. We then checked the initial alignments by eye and adjusted them slightly. Phylogenetic trees were constructed by using maximum likelihood ML) and Bayesian inference (BI). Prior to ML and Bayesian analyses, we chose the optimum substitution models for entire sequences using KAKUSAN 4 (Tanabe 2011) based on the Akaike information criterion (AIC). The best model selected for ML and BI was the general time-reversible model (GTR: Tavaré 1986) with a gamma shape parameter (G: 0.337 in ML and 0.376 in BI). For ML analysis, the TREEFINDER software (Jobb et al. 2004) was used while BI analysis was conducted using MRBAYES v. 3.2.7a (Ronquist et al. 2012). The strength of nodal support in the ML tree was analyzed using non-parametric bootstrapping (MLBS) with 1,000 replicates. We regarded tree nodes in the ML tree with bootstrap values of 75% or greater as sufficiently resolved (Hillis and Bull 1993), and nodes with a BPP of 95% or greater as significant in the BI analysis (Leaché and Reeder 2002). The BI summarized two independent runs of four Markov Chains for 10,000,000 generations. A tree was sampled every 100 generations and a consensus topology was calculated for 70,000 trees after discarding the first 30,001 trees (burn-in 1,000,000). We checked parameter estimates and convergence using TRACER v. 1.6 (Rambaut and Drummond 2013). Pairwise comparisons of uncorrected sequence divergences (p-distance) were calculated with MEGA 7 (Kumar et al. 2016) for ND2 fragments only between species of the genus *Tylototriton*. Variance was estimated using bootstrap method with 1,000 replicates using nucleotide substitution while gap/missing data were treated via pairwise deletion.

### ﻿Morphological characters

A total of 27 morphological characters were measured following Bernardes et al. (2020) to the nearest 0.01 mm with a digital caliper as follows: anterior snout-vent length (**SVL**) from tip of snout to anterior tip of vent ; head length (**HL**) ; head width (**HW**) measured behind the eyes and before the beginning of the parotoids ; maximum head width (**MHW**); parotoid length (**PL**) ; parotoid width (**PW**) ; maximum parotoid height (**PH**) ; eye length (**EL**) ; eye-narial distance (**EN**) ; inter-narial distance (**IN**) ; inter-eye distance (**IE**) ; lower jaw length (**LJL**) from tip of lower jaw to jaw angle ; maximum upper eyelid length (**UEL**) ; humerus length (**HUM**) ; radius length (**RAD**) ; femur length (**FEM**) ; tibia length (**TIB**) ; total forelimb length (**FORE**) ; total hindlimb length (**HIND**) ; tail length (**TL**) from anterior of vent to tail tip ; tail height (**TH**) ; cloaca length (**CIL**): length of cloaca muscle ; cloaca width (**CIW**) ; width of vertebral cord measured at the height of the 5^th^ nodule (**WVr**) ; length of the 5^th^ anterior dorsal nodule (**L5W**) ; axilla to groin (**AG**) ; trunk length from wrinkle of throat to anterior tip of vent (**TkL**) ; total length (**TOL**).

Morphological comparisons between the new taxon and its congeners were based on the specimens examination and the following literature: Fei et al. (1984), Nussbaum et al. (1995), Böhme et al. (2005), Chen et al. (2010), Stuart et al. (2010), Hou et al. (2012), Shen et al. (2012), Nishikawa et al. (2013a,b), Nishikawa et al. (2014), Yang et al. (2014), Khatiwada et al. (2015), Le et al. (2015), Phimmachak et al. (2015), Fei and Ye (2016), Hernandez (2016), Qian et al. (2017), Grismer et al. (2018, 2019), Zaw et al. (2019), Bernardes et al. (2017a, 2020), Pomchote et al. (2020, 2021), Lyu et al. (2021), Poyarkov et al. (2021a), Li et al. (2022), and Luo et al. (2022).

## ﻿Results

### ﻿Phylogenetic analyses

**Figure 1.**
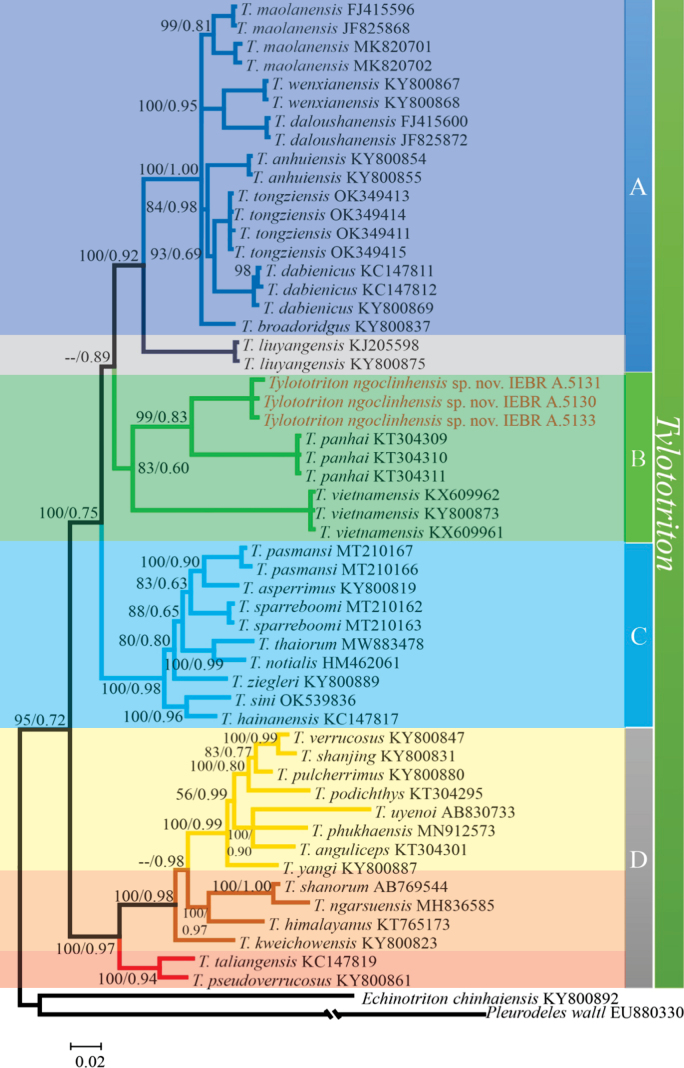
BI tree from a 1035 bp sequence of the mitochondrial ND2 gene for *Tylototriton* and outgroup species; ML inferences bootstrap support value (ML-BS) and Bayesian posterior probabilities (BPP) are shown near the node. For GenBank accession numbers, refer to Table 1.

Aligned, combined sequences yielded a total of 1,035 characters. Of 1,035 nucleotide sites, 416 were variable and 336 were parsimony informative within the in-group. The ML and Bayesian analyses produced topologies with -lnL = 7442.0236 and 7521.862, respectively. Phylogenetic analyses employing ML and BI methods yielded slightly different topologies only among referenced species, and only the ML tree is presented in Fig. 1.

Monophyly of *Tylototriton* with respect to the outgroup species was fully supported (each 100% support in ML bootstrap value and Bayesian posterior probability) and samples were split into four major clades named A, B, C, and D.

Monophyly of clade A, including *T.maolanensis* Li, Wei, Cheng, Zhang & Wang, 2020; *T.wenxianensis* Fei, Ye & Yang, 1984; *T.anhuiensis* Qian, Sun, Li, Guo, Pan, Kang, Wang, Jiang, Wu & Zhang, 2017; *T.dabienicus* Chen, Wang & Tao, 2010; *T.tongziensis* Li, Liu, Shi, Wei & Wang, 2022; *T.broadoridgus* Shen, Jiang & Mo, 2012 *T.daloushanensis* Zhou, Xiao, and Luo, 2022; and *T.liuyangensis* Yang, Jiang, Shen, and Fei, 2014, was well supported (100% and 92% support).

The undescribed species of *Tylototriton* from Kon Tum Province, Vietnam was clustered in clade B with *T.panhai* Nishikawa, Khonsue, Pomchote & Matsui, 2013 from Laos and *T.vietnamensis* from Vietnam, but the support was not significant, particularly for Bayesian posterior probability (0.83 and 60%) (Table 2).

**Table 2.** Mean uncorrected (p) distance (%) among 1,035 bp fragments of ND2 of the genus *Tylototriton* and related taxa.

		1.	2.	3.	4.	5.	6.	7.	8.	9.	10.	11.	12.	13.	14.	15.	16.	17.	18.	19.	20.	21.	22.	23.	24.	25.	26.	27.	28.	29.	30.	31.	32.
1.	*Tylototritonngoclinhensis* sp. nov.	0.00–0.31																															
2.	*T.anguliceps*	10.08–11.55	-																														
3.	*T.anhuiensis*	10.19–10.64	10.82	-																													
4.	*T.asperrimus*	9.88–10.64	11.30	8.31	-																												
5.	*T.broadoridgus*	9.54–9.73	10.92	3.67	8.50	-																											
6.	*T.dabienicus*	9.23–10.03	11.88–12.08	3.86–3.96	8.99–9.08	3.38–3.48	0.10–0.29																										
7.	*T.daloushanensis*	9.54–9.73	11.69	4.64	9.28	4.25	4.44–4.45	-																									
8.	*T.hainanensis*	7.72–8.21	9.95	7.83	5.02	7.63	8.79–8.89	9.18	-																								
9.	*T.himalayanus*	8.64–9.12	6.86	10.82	11.50	10.43	11.01–11.21	11.01	9.76	-																							
10.	*T.kweichowensis*	8.64–9.12	6.18	9.86	10.14	8.99	10.24–10.34	10.63	8.60	5.31	-																						
11.	*T.liuyangensis*	8.62–9.12	10.14–10.24	7.25–7.34	8.70–8.79	7.05–7.15	7.25–7.44	7.44–7.54	7.73–7.83	10.05–10.14	9.47–9.57	0.10																					
12.	*T.maolanensis*	9.57–11.55	10.72–11.40	3.48–3.57	8.60	2.80–3.00	3.38–3.67	4.35–4.44	8.21–8.31	10.63–11.11	9.66–9.95	7.05–7.25	0.00–2.13																				
13.	*T.ngarsuensis*	12.04–12.36	7.74	12.05	12.34	11.56	12.34–12.54	12.44	10.97	6.46	6.95	11.36–11.46	11.46–11.85	-																			
14.	*T.notialis*	9.26–9.73	10.63	8.50	4.93	8.41	9.18–9.28	9.47	4.83	10.63	9.86	8.70–8.79	8.60–8.70	11.75	-																		
15.	*T.panhai*	6.77–6.99	12.95	9.57	11.11	8.37	9.47–9.57	10.24	9.66	12.27	10.72	9.37–9.47	9.86–10.14	13.22	10.34	0.00																	
16.	*T.pasmansi*	9.57–10.33	10.40–10.69	8.41–8.50	3.38–3.48	8.31–8.41	9.28–9.37	9.57–9.66	4.83	11.21–11.69	9.95–10.24	8.31–8.41	8.89–9.08	12.44–12.93	4.64–4.93	10.43–10.53	0.48																
17.	*T.phukhaensis*	11.11–11.38	4.29	11.40	11.99	11.11	11.89–12.09	12.28	10.62	7.02	6.34	10.33–10.43	11.11–11.89	9.01	11.01	12.87	12.09–12.38																
18.	*T.podichthys*	9.88–10.15	5.31	9.86	10.82	9.95	10.63–10.82	10.14	10.14	6.76	6.09	10.05–10.14	10.05–10.43	8.33	10.72	12.27	11.21–11.50	6.24															
19.	*T.pseudoverrucosus*	9.26–9.54	8.50	9.47	9.66	9.28	9.47–9.66	9.66	8.50	7.05	5.99	9.80–9.18	9.28–9.86	8.72	9.28	10.43	9.37–9.86	8.38	7.25														
20.	*T.pulcherrimus*	9.73–10.15	3.96	9.55–9.95	10.92	9.86	10.34–10.53	12.24	9.47	6.18	5.41	9.76–9.86	9.76–10.14	6.95	10.24	12.08	10.50–11.20	4.39	3.67	6.67													
21.	*T.shanjing*	11.25–11.69	4.35	10.43	11.40	10.24	10.82–11.01	10.63	10.24	6.28	5.99	10.53–10.63	9.95–10.72	7.35	11.01	12.66	12.08–11.79	5.36	4.25	7.73	2.51												
22.	*T.shanorum*	9.88–10.33	6.67	10.82	11.30	10.14	10.92–11.11	11.40	9.76	5.02	5.89	10.24–10.34	10.24–10.63	1.76	10.63	12.37	11.21–11.69	7.60	7.44	7.92	6.18	6.57											
23.	*T.sini*	7.72–8.81	9.57	8.21	5.22	8.60	8.99–9.08	8.79	3.19	9.47	8.89	7.83–7.92	8.31–8.6	10.77	5.51	10.34	5.12–5.60	10.62	9.66	8.50	9.28	10.24	9.57										
24.	*T.sparreboomi*	8.64–10.03	10.72–10.92	7.92–8.12	4.15	8.02–8.21	8.50–8.70	9.28–9.47	4.44–4.64	10.53–10.72	9.57–9.76	8.60–8.79	8.50–8.79	11.95–12.14	4.73	9.95–10.14	3.96–4.25	11.31–11.50	10.92–11.11	9.08–9.28	10.34–10.53	11.40–11.59	10.53–10.72	4.83–5.02	0.19								
25.	*T.taliangensis*	9.42–9.85	8.41	8.99	9.28	9.28	9.08–9.28	9.47	8.60	7.54	6.47	8.99–9.08	8.89–9.57	9.11	9.47	10.53	9.76–10.05	8.58	7.73	2.71	7.25	7.34	8.12	8.79	9.28–9.47								
26.	*T.thaiorum*	8.64–9.12	10.72	8.21	5.31	7.92	8.70–8.79	8.99	4.64	10.53	9.95	8.50–8.60	8.12–8.21	12.05	2.80	10.24	4.64–4.93	10.82	10.72	8.89	10.24	11.01	10.92	5.89	4.64–4.83	9.08							
27.	*T.tongziensis*	8.33–9.12	10.05–10.14	2.80–2.90	7.83–7.92	2.61–2.71	2.61–2.80	3.67	7.44–7.54	9.95–10.05	8.99–9.08	6.47–6.67	2.42–2.80	10.87–10.97	7.92–8.02	9.18–9.28	8.12–8.21	10.43–10.53	9.37–9.47	8.12–8.21	9.08–9.18	9.86–9.95	9.66–9.76	7.44–7.54	7.73–8.02	8.02–8.12	7.44–7.54	0.00–0.10					
28.	*T.uyenoi*	12.96–13.23	7.25	12.75	12.85	12.75	13.04–13.14	13.24	12.46	8.50	8.02	12.56–12.66	12.56–12.75	10.09	13.04	14.40	13.04–13.33	7.21	8.21	9.95	6.76	7.25	9.08	12.27	13.62–13.82	9.95	12.66	11.59–11.69					
29.	*T.verrucosus*	10.94–11.08	4.35	10.63	11.50	10.24	10.82–11.01	10.82	10.24	6.47	5.70	10.24–10.34	9.95–10.72	6.95	11.01	12.56	11.79–12.08	4.97	4.06	7.54	2.13	1.16	6.18	10.05	11.40–11.59	7.44	11.01	9.86–9.95	7.25				
30.	*T.vietnamensis*	11.11–11.38	13.04–13.17	10.64–10.84	10.82–11.04	10.84–10.92	10.44–10.92	11.14–11.21	10.72–10.94	12.36–12.75	11.65–11.98	10.64–10.94	10.53–11.14	12.97–13.17	11.35–11.55	10.54–11.24	11.59–12.75	13.48–13.74	12.95–13.07	11.45–11.65	11.79–12.06	12.57–12.66	11.85–12.06	11.01–11.45	10.54–10.82	10.94–11.14	11.55–11.75	9.83–10.14	14.78–14.99	12.08–12.06	0.00		
31.	*T.wenxianensis*	9.88–10.33	10.72	4.15	8.70	4.25	4.73–4.83	3.77	8.70	10.53	9.76	6.96–7.05	4.15–4.25	12.05	8.89	10.14	9.47–9.57	11.11	9.57	9.28	9.08	9.86	11.11	9.08	9.08–9.28	8.60	8.60	3.57–3.67	12.46	9.66	10.23–10.24	0.00	
32.	*T.yangi*	11.73–12.00	4.15	10.14	10.14	10.05	10.82–11.01	10.43	9.57	6.57	6.18	9.37–9.47	9.57–10.34	7.35	10.24	12.46	10.53–10.82	5.56	5.12	7.44	3.77	4.35	6.38	8.79	9.95–10.14	7.83	10.34	9.18–9.28	7.54	3.96	11.96–12.16	9.66	
33.	*T.ziegleri*	8.95–9.73	10.82	8.12	4.44	7.92	8.79–8.89	9.28	4.35	10.92	9.18	8.41–8.50	8.41–8.50	12.05	4.93	9.95	4.83–5.12	11.21	10.72	9.28	10.43	11.21	10.92	5.22	4.35	9.47	4.83	7.54–7.63	13.33	11.21	10.94–11.21	8.79	10.05

Monophyly of clade C, including *T.pasmansi*; *T.sparreboomi*; *T.asperrimus* Unterstein, 1930; *T.thaiorum*; *T.notialis* Stuart, Phimmachak, Sivongxay & Robichaud, 2010; *T.sini* Lyu, Wang, Zeng, Zhou, Qi, Wan & Wang, 2021; *T.hainanensis* Fei, Ye & Yang, 1984 and *T.ziegleri*, was well supported (100% and 98% support).

Monophyly of clade D, including *T.verrucosus* Anderson, 1871; *T.shanjing* Nussbaum, Brodie & Yang, 1995; *T.podichthys* Phimmachak, Aowphol & Stuart, 2015; *T.pulcherrimus* Hou, Zhang, Li & Lu, 2012; *T.uyenoi* Nishikawa, Khonsue, Pomchote & Matsui, 2013; *T.phukhaensis* Pomchote, Khonsue, Thammachoti, Hernandez, Peerachidacho, Suwannapoom, Onishi & Nishikawa, 2020; *T.anguliceps*; *T.yangi* Hou, Zhang, Zhou, Li & Lu, 2012; *T.shanorum* Nishikawa, Matsui & Rao, 2014; *T.ngarsuensis* Grismer, Wood, Quah, Thura, Espinoza, Grismer, Murdoch & Lin, 2018; *T.himalayanus* Khatiwada, Wang, Ghimire, Vasudevan, Paudel & Jiang, 2015; *T.kweichowensis* Fang & Chang, 1932; *T.taliangensis* Liu, 1950 and *T.pseudoverrucosus* Hou, Gu, Zhang, Zeng & Lu, 2012, was also strongly supported (100% and 97% support).

There is a clear genetic distance between clade A and the remaining clades B, C, and D: from 8.33% (*Tylototriton* sp. from Kon Tum Province, Vietnam and *T.tongziensis*) to 11.55% (*Tylototriton* sp. from Kon Tum Province, Vietnam and *T.maolanensis*); from 7.44% (*T.sini* and *T.tongziensis*) to 9.66% (*T.pasmansi* and *T.daloushanensis*); from 8.02 (*T.daloushanensis* and *T.uyenoi*) to 13.24% (*T.tongziensis* and *T.taliangensis*), respectively. The genetic distance between clade B and the two clades C and D ranges from 7.72% (*Tylototriton* sp. from Kon Tum Province, Vietnam and *T.thaiorum*) to 11.79% (*T.vietnamensis* and *T.thaiorum*); from 8.64% (*Tylototriton* sp. from Kon Tum Province, Vietnam and *T.pseudoverrucosus*) to 14.99% (*T.vietnamensis* and *T.uyenoi*) and clear genetic distances between clades C and D: from 8.5% (*T.hainanensis* and *T.pseudoverrucosus*) to 13.82 (*T.sparreboomi* and *T.uyenoi*).

The *Tylototriton* population from Kon Tum Provice, Vietnam exhibited distinct genetic distances from the thirty-three examined species of *Tylototriton*, with uncorrected *p*-distance of 6.77% (compared to *T.panhai* from Laos) to 12.36% (compared to *T.ngarsuensis*), being higher than that between some pairs of sister species, for example, *T.verrucosus* vs *T.shanjing* (1.16%), and *T.maolanensis* vs *T.tongziensis* (2.42%).

Furthermore, the population of *Tylototriton* sp. from Kon Tum Province, Vietnam was also clearly separated morphologically from all the other congeners, including its sister species *T.panhai*, which is in congruence with the genetic separation. Thus, we describe the new *Tylototriton* population from Kon Tum Province, Vietnam as a new species.

## ﻿Taxonomy

### ﻿ Tylototriton ngoclinhensis sp. nov.

Taxon classificationAnimaliaCaudataSalamandridae

539AFD20-DB86-5810-A95D-C8F87CDA2D03

https://zoobank.org/CBC407D7-E692-4363-A2BA-EECA307C7AC9

**Figure 2.**
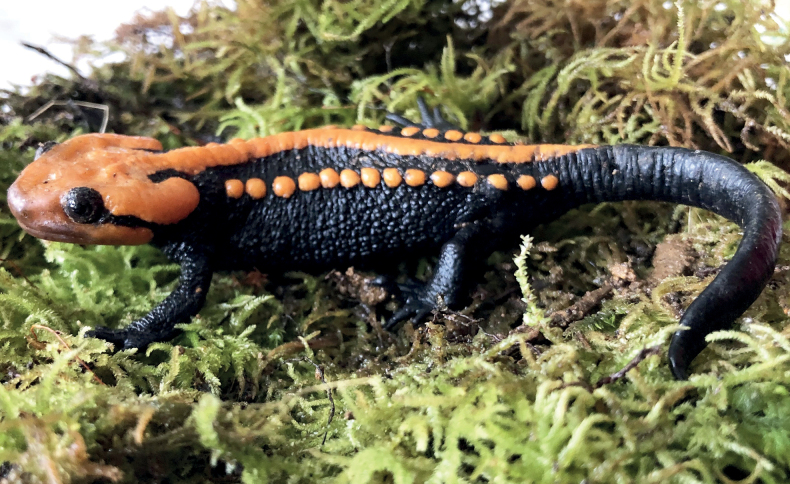
Dorsolateral view of *Tylototritonngoclinhensis* sp. nov., holotype male (IEBR A.5130), in life.

**Figure 3.**
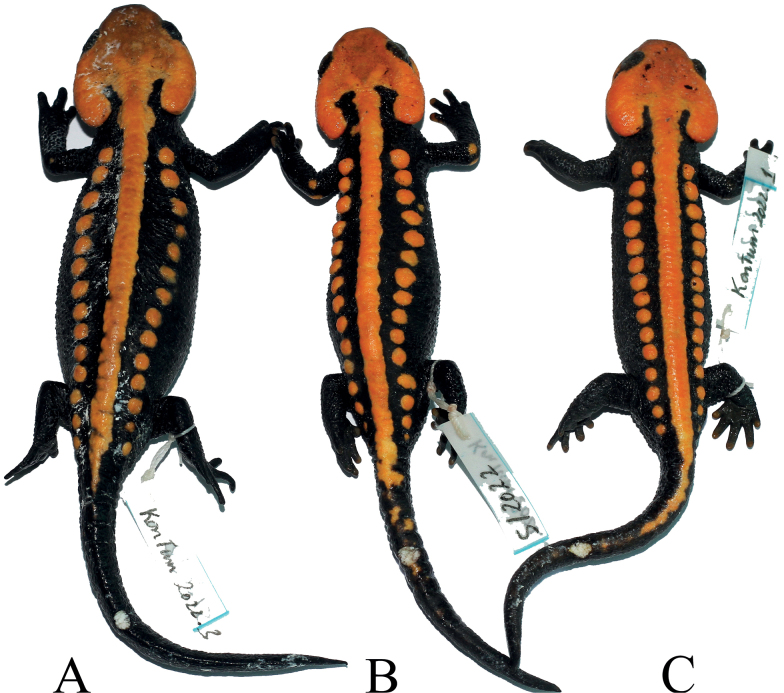
Dorsal views of the holotype **B** of *Tylototritonngoclinhensis* sp. nov. (IEBR A.5130, male) and two paratypes **A** (IEBR A.5133, female) and **C** (IEBR A.5131, male) in preservative.

**Figure 4.**
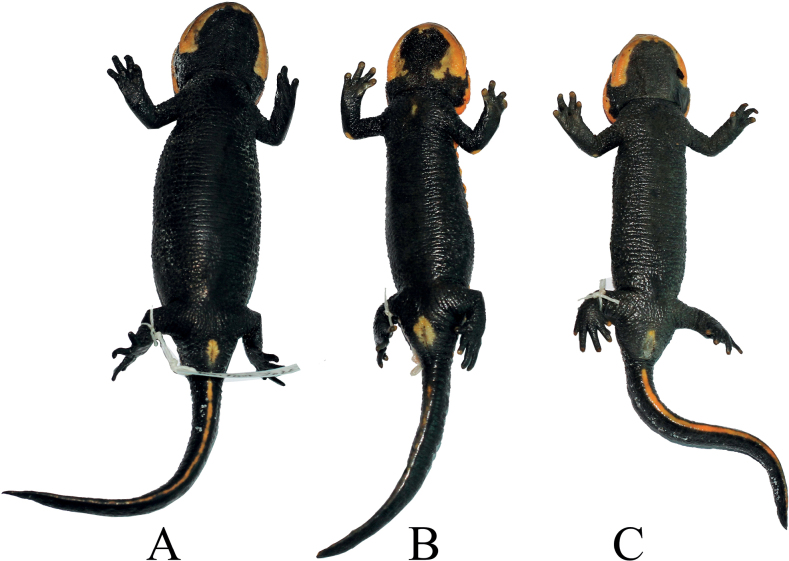
Ventral views of the holotype **B** of *Tylototritonngoclinhensis* sp. nov. (IEBR A.5130, male) and two paratypes **A** (IEBR A.5133, female) and **C** (IEBR A.5131, male) in preservative.

**Figure 5.**
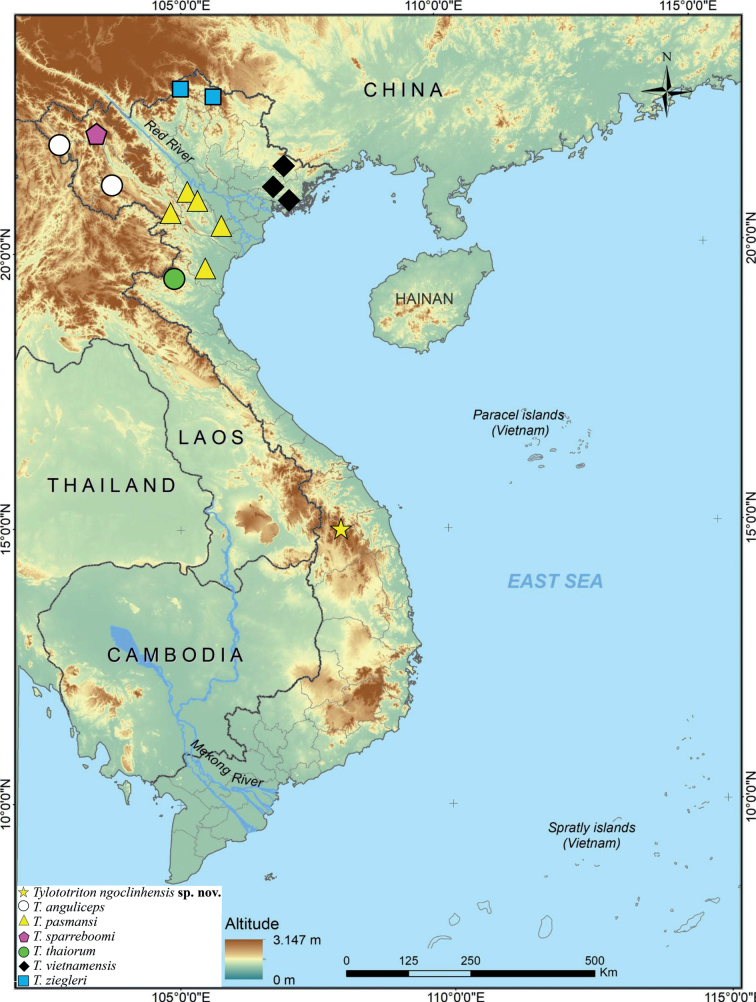
Type locality of *Tylototritonngoclinhensis* sp. nov. (yellow star) and congeners; elevations (from 0-1200+ m) increasing from green to brown (Tran Anh Tuan, after Sterling et al. 2006).

**Figure 6.**
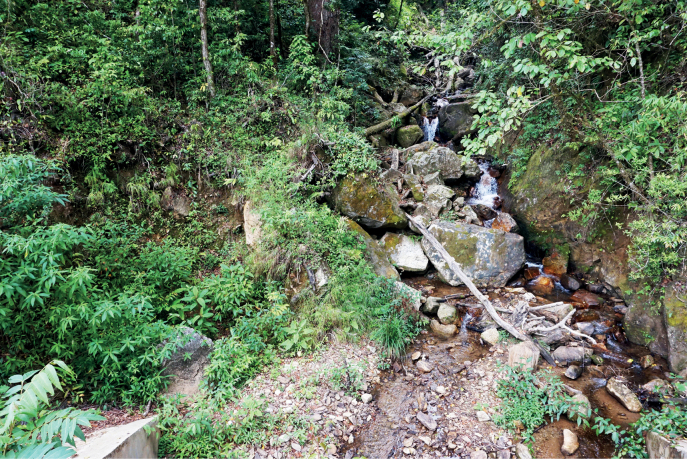
Habitat at the type locality of *Tylototritonngoclinhensis* sp. nov. on Ngoc Linh Mountain.

**Figure 7.**
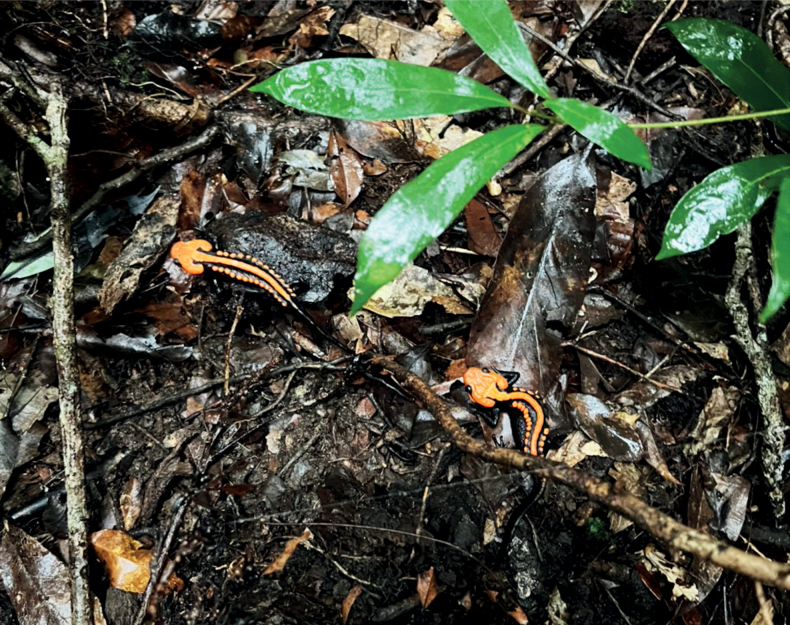
*Tylototritonngoclinhensis* sp. nov. in its microhabitat at the type locality.

**Figure 8.**
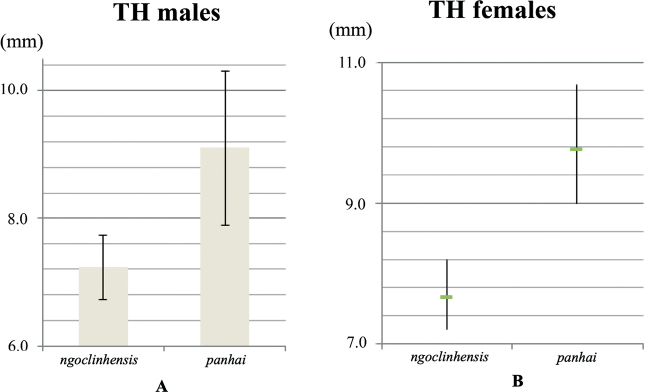
Comparison of average tail heights (mm) between *Tylototritonngoclinhensis* sp. nov and *T.panhai*. The left side shows data for males (**A**) of *Tylototritonngoclinhensis* sp. nov. (*n* = 3; own data) and *T.panhai* (*n* = 15; taken from Phimmachak et al. 2015), showing 2 standard deviation (2SD) bars, which represent 95% of the population. The right side shows data for females (**B**) of *Tylototritonngoclinhensis* sp. nov. (*n* = 3; own data) and *T.panhai* (*n* = 3) including two data points from Phimmachak et al. (2015), and one from Nishikawa et al. (2013a); averages marked by green horizontal lines, and ranges as vertical lines.

Figs 2 , 3 , 4 , 5 , 6 , 7 , 8 Proposed common name: Ngoc Linh Crocodile Newt

#### Material examined.

***Holotype*.**IEBR A.5130 (Field No KT 2022.02), an adult male collected by T. M. Phung on 22 May 2022 in the montane evergreen forests of Ngoc Linh Natural Reserve, Dak Glei District, Kon Tum Province, Central Vietnam at 1.854 m a.s.l. ***Paratypes*.**IEBR A.5131, A.5132 (Field No KT 2022.01, KT 2022.5), two adult males and IEBR A.5133, A.5134 (Field No KT2022.03, KT 2022.6), two adult females, collected by T. M. Phung; IEBR A.5135 (Field No KT 2022.4), an adult female, collected by S. T. Le on 20 May 2022, the same collection data as the holotype.

#### Etymology.

The specific epithet *ngoclinhensis* refers to the type locality of the new species, Ngoc Linh Mountain in the Central Highlands of Vietnam. As common names, we suggest Ngoc Linh Crocodile Newt (English), Cá cóc sần ngọc linh (Vietnamese).

#### Diagnosis.

The new species is assigned to the genus *Tylototriton* and the subgenus Yaotriton based on the results of the molecular phylogenetic analyses and the following combination of morphological attributes: rough skin covered with fine warts, the presence of dorsolateral bony ridges on the head; the presence of dorsolateral glandular warts on the body; quadrate spine absent (Nussbaum and Brodie 1982; Nishikawa et al. 2013a; Le et al. 2015). Furthermore, the species is diagnosed from its congeners by the following combination of morphological characters: (1) size medium (SVL 60.8–66.5 mm, TL 57.6–61.8 mm in males, and SVL 72.5–75.6 mm, TL 62.9–67.9 mm in females); (2) head longer than wide; (3) parotoids very prominent and enlarged, projecting backwards; (4) tail length shorter than the snout-vent length; (5) vertebral ridge large, high, and glandular in appearance (6) 14 distinct dorsolateral glandular warts; (7) gular fold present; (8) tips of fore- and hind limbs overlapping when adpressed along the body; (9) tips of fingers reaching between eye and nostril when foreleg is laid forward; (10) dorsal surface and lateral sides of the head, upper and lower lips, rib nodules, vertebral ridge, peripheral area of the cloaca, and the ventral edge of tail with orange coloration; (11) presence of a distinct black line extending from the posterior end of the eye towards the shoulder.

#### Description of holotype.

A medium-sized male (SVL 66.5 mm, TL 59.5 mm). Head longer than wide (HW/HL 81.6%); head slightly concave on the top; snout short, truncate in dorsal view, slightly angular shaped in profile and protruding beyond lower jaw; nostril closer to the snout tip than to the eye; upper lip thick, fleshy and overlapping lower lip under the eye region; dorsolateral bony ridges on head prominent, moderately protruding, from above eye to above anterior end of parotoid, posterior ends relatively thick and scrolled inside; mid-dorsal ridge on head distinct and thin; parotoids enlarged, projecting backwards; tongue oval, attached to anterior floor of mouth, free laterally and posteriorly; vomerine teeth series in an inverted V-shape, converging anteriorly and reaching choanae; glandular vertebral ridge large, high, segmented tuberculate, extending from top of head to base of tail; rib nodules large, forming knob-like warts, distinctly isolated from each other, 14 on each side of body from axilla to base of tail; gular fold present.

Limbs comparatively long, and slender; length of forelimbs approximately equal to hind limbs; relative length of forelimb FORE/SVL ratio 39.0%, relative length of hind limb HIND/SVL ratio 38.1%; tips of forelimb and hind limb overlapping when adpressed along the body; tips of fingers reaching between eye and nostril when foreleg is laid forward; fingers and toes well developed, free of webbing; fingers four, comparative finger lengths 1FL<4FL<2FL<3FL; toes five, comparative toe lengths 1TL<5TL<2TL<4TL<3TL.

Tail length shorter than the snout-vent length (TL/SVL 89.5%); tail compressed laterally, the base relatively broad, tapering posteriorly, tail tip pointed; tail height less than the width at the tail base; dorsal fin fold relatively high; ventral side smooth. In general, the appearance of the tail is relatively low and flat.

Dorsal skin very rough, with small granules and larger warts on dorsal surfaces of head and dorsum, lateral sides of body and tail; ventral skin with tubercles shaped like transverse wrinkles; throat with numerous tiny flat tubercles; surfaces of head ridges and middorsal vertebral ridge rough; limbs dorsally with numerous tiny tubercles, volar and plantar surfaces of hands and feet with tiny grooves forming reticulated pattern; flattened outer metacarpal and metatarsal tubercles distinct on palms and soles, respectively. Cloacal region slightly swollen, vent as a longitudinal slit, vent edges with numerous small transverse folds.

#### Coloration in life.

In life, ground color of dorsal and ventral surfaces black; dorsal surface and lateral sides of head and lower lips to jaw angles, rib nodules, vertebral ridge, the peripheral area of the cloaca and the ventral edge of the tail orange; tips of fingers, toes and elbow orange-brown.

#### Coloration in preservative.

The specimen in preservative is blackish brown. The orange coloration in life has faded to pale yellow.

#### Secondary sexual characteristics.

Males are probably smaller than females but sample size was small (*n* = 3) and thus needs confirmation based on further records in the future. The female cloacal slit is short and its inner cloacal walls have no papilla. The male has papilla on its inner cloacal wall and its cloaca presents a long slit.

#### Distribution.

The new species is currently known only from the Ngoc Linh Nature Reserve, Kon Tum Province, in the Central Highlands of Vietnam (Fig. 5).

#### Ecological notes.

All specimens were collected during the day on the forest floor, under rotten trees or under moss, near a small rocky stream (Figs 6, 7). The surrounding habitat at the type locality of the new species in Ngoc Linh Mountain was primary montane evergreen broadleaf forest, at elevations between 1,800 and 2,300 m asl.

#### Morphological measurements.

Morphometric measurements of *Tylototritonngoclinhensis* sp. nov. examined in this study are given in Table 3.

**Table 3.** Morphometric measurements (mm) of *Tylototritonngoclinhensis* sp. nov. examined in this study.

Voucher	IEBR A.5130 Holotype	IEBR A.5131 Paratype	IEBR A.5132 Paratype	IEBR A.5133 Paratype	IEBR A.5134 Paratype	IEBR A.5135 Paratype
Sex	♂	♂	♂	♀	♀	♀
**SVL**	**66.5**	**65.7**	**60.8**	**75.6**	**74.8**	**72.5**
**HL**	20.6	19.9	18.9	23.8	22.2	21.5
**HW**	16.8	15.6	16.3	18.6	17.5	17.4
**MHW**	18.5	18.4	17.9	20.1	19.5	19.4
**PL**	11.5	11.4	11.2	13.5	12.1	12.4
**PW**	5.7	5.8	5.3	5.9	5.9	5.8
**PH**	5.9	6.3	5.2	6.5	6.2	6.1
**EL**	4.8	4.4	4.4	5.2	4.9	4.9
**EN**	4.2	4	3.9	4.8	4.6	4.5
**IN**	5.5	5.7	5.4	6.4	6.6	6.2
**IE**	8.4	8.2	8.5	9.8	9.5	9.4
**LJL**	14.1	13.6	13.7	17.1	16.1	16.6
**UEL**	2.5	2.6	2.2	2.8	2.5	2.6
**HUM**	9.2	8.4	8.7	9.5	9.4	9.3
**RAD**	16.7	15.6	15.8	17.6	17.3	17.2
**FEM**	8.7	8.2	8.3	8.8	8.9	8.6
**TIB**	16.6	15.9	16.8	17.8	18.1	17.2
**FORE**	25.9	24	24.5	27.1	26.7	26.5
**HIND**	25.3	24.1	25.1	26.6	27	26.2
**TL**	**59.5**	**57.6**	**61.8**	**67.9**	**62.9**	**66.2**
**TH**	7.2	7	7.5	8.2	7.2	7.6
**CIL**	7.4	6.9	6.7	5.9	5.5	5.8
**CIW**	4.9	4.6	4.3	3.7	3.5	3.5
**WVr**	4.2	4.5	3.8	4.4	4.2	4.1
**L5W**	3.2	3.1	2.7	3.5	3.3	3.4
**AG**	35.4	34.5	31.7	41.4	39.4	37.8
**TkL**	49.7	49.3	45.5	58.7	55.7	53.8
**ToL**	**126**	**123.3**	**122.6**	**143.5**	**137.7**	**138.7**

#### Morphological comparisons.

We compared the new species with other members of the genus *Tylototriton* based on data obtained from the literature (Table 4).

**Table 4.** Morphological comparisons between *Tylototritonngoclinhensis* sp. nov. with other members of the subgenus Yaotriton (morphological data obtained from Fei et al. (1984), Nussbaum et al. (1995), Böhme et al. (2005), Chen et al. (2010), Stuart et al. (2010), Hou et al. (2012), Shen et al. (2012), Nishikawa et al. (2013a,b), Yang et al. (2014), Phimmachak et al. (2015), Fei and Ye (2016), Hernandez (2016), Qian et al. (2017), Bernardes et al. (2017a, 2020), Li et al. (2020), Lyu et al. (2021), Poyarkov et al. (2021a), Li et al. (2022), Luo et al. (2022). Abbreviations are as follows: TOL = total length, / = characters unobtainable from literature.

Species	TOL of males	TOL of females	Gular fold	Reach of finger tips when forelimbs stretched forward	Reach of tips of fore-and hind limbs when adpressed along body	Rib nodules shape	Vertebral ridge	Orange markings on the parotoid	Orange coloration on the rib nodules
*Tylototritonngoclinhensis* sp. nov.	122.6–126.0	137.7–143.5	present	between the eye and nostril	overlapping	knob-like	segmented tuberculate	present	present
*T.anhuiensis*	118.9–145.7	103.8–165.4	present	/	meeting	slightly flattened	not segmented	absent	absent
*T.asperrimus*	118.0-138.0	149.0–202.0	present	reaching to the nostril or eye	meeting or overlapping	knob-like	not segmented	absent	absent
*T.broadoridgus*	110.4–140.3	138.9–162.5	absent	anterior corner of eye	not touched	slightly flattened	not segmented	absent	absent
*T.dabienicus*	120.3–135.3	134.9–155.5	present	anterior corner of eye	not touched	slightly flattened	not segmented	absent	absent
*T.daloushanensis*	/	/	present	eyes to nostrils	overlapping	slightly flattened	not segmented	absent	absent
*T.hainanensis*	137.0–148.0	125.0–140.0	present	eye	meeting or overlapping	slightly flattened	not segmented	absent	absent
*T.liuyangensis*	110.1–146.5	138.6–154.2	present	eye	not touched	slightly flattened	not segmented	absent	absent
*T.lizhenchangi*	145.6–173.0	150.0–156.5	present	Tip of snout	overlapping	slightly flattened	not segmented	absent	absent
*T.maolanensis*	151.0–172.0	142.7–170.5	present	beyond the snout	overlapping	knob-like	not segmented	absent	absent
*T.notialis*	109.1–130.4	141.8	present	/	/	knob-like	not segmented	absent	present
*T.panhai*	129.9–1603	160.0–166.8	present	/	/	knob-like	not segmented	present	present
*T.pasmansi*	/	160.0	present	eye	/	knob-like	not segmented	absent	absent
*T.sini*	118.4–124.5	144.5	present	/	overlapping	knob-like	not segmented	absent	absent
*T.sparreboomi*	/	/	present	nostril	/	knob-like	not segmented	absent	absent
*T.thaiorum*	116.1–138.0	/	present	/	overlapping	knob-like, in irregular series	not segmented	absent	absent
*T.tongziensis*	120.5–135.1	123.5–127.6	present	beyond the snout	overlapping	slightly flattened	not segmented	absent	absent
*T.vietnamensis*	113.0–121.8	/	absent	/	/	slightly flattened	not segmented	absent	absent
*T.wenxianensis*	126.0–133.0	/	present	nostril	meeting or overlapping	slightly flattened	not segmented	absent	absent
*T.ziegleri*	/	/	present	/	overlapping	knob-like	segmented tuberculate	absent	absent

*Tylototritonngoclinhensis* sp. nov. differs from *T.anhuiensis* by different shape of dorsolateral glandular warts (knob-like vs slightly flattened), the presence of orange markings on the parotoids (vs absent), the presence of orange coloration on the dorsolateral glandular warts (vs absent), and tips of fore-and hind limbs overlapping when adpressed along the body (vs meeting); from *T.asperrimus* by having a smaller size in females (TOL 137.7–143.5 mm vs 149.0–202.0 mm), head longer than wide (vs head wider than long), the presence of orange markings on the parotoids (vs absent), and the presence of orange coloration on the dorsolateral glandular warts (vs absent); from *T.broadoridgus* by different shape of dorsolateral glandular warts (knob-like vs slightly flattened), the presence of orange markings on the parotoids (vs absent), the presence of orange coloration on the dorsolateral glandular warts (vs absent), and tips of fore and hind limbs overlapping when adpressed along the body (vs separated from each other); from *T.dabienicus* by different shape of dorsolateral glandular warts (knob-like vs slightly flattened), the presence of orange markings on the parotoids (vs absent), the presence of orange coloration on the dorsolateral glandular warts (vs absent), tips of fore-and hind limbs overlapping when adpressed along the body (vs separated from each other), and finger tips reaching between eye and nostril when foreleg is laid forward (vs reaching anterior corner of eye); from *T.daloushanensis* by different shape of dorsolateral glandular warts (knob-like vs slightly flattened), the presence of orange markings on the parotoids (vs absent), and the presence of orange coloration on dorsolateral glandular warts (vs absent); from *T.hainanensis* by having a smaller size in males (TOL 122.6–126.0 mm vs 137.4–148.0 mm), head longer than wide (vs head wider than long), different shape of dorsolateral glandular warts (knob-like vs slightly flattened), the presence of orange marking on the parotoids (vs absent), the presence of orange coloration on the dorsolateral glandular warts (vs absent), and finger tips reaching between eye and nostril when foreleg laid forward (vs reaching eye); from *T.joe* by having larger size (TOL 122.6–126.0 mm vs 108–115 mm in males and 137.7–143.5 mm vs 121–128 mm in females), ventral edge of the tail orange, and tip of fingers, toes and elbow orange-brown (vs whole body dark brown but brownish tip of toes and tip of tail in some individuals); from *T.liuyangensis* by different shape of dorsolateral glandular warts (knob-like vs slightly flattened), the presence of orange markings on the parotoids (vs absent), the presence of orange coloration on the dorsolateral glandular warts (vs absent), tips of fore-and hind limbs overlapping when adpressed along body (vs separated from each other), and finger tips reaching between eye and nostril when foreleg laid forward (vs reaching eye); from *T.lizhenchangi* by having a smaller size (TOL 122.6–126.0 mm vs 145.6–173.0 mm in males, TOL 137.7–143.5 mm vs 150.0–156.5 mm in females), different shape of dorsolateral glandular warts (knob-like vs slightly flattened), the presence of orange coloration on the dorsolateral glandular warts (vs absent), tips of fingers reaching between eyes and nostrils when foreleg laid forward (vs reaching to tip of snout), and the presence of orange markings on the parotoids (vs absent); from *T.maolanensis* by having a smaller size (TOL 122.6–126.0 mm vs 151.0–172.0 mm in males, TOL 137.7–143.5 mm vs 142.7–170.5 mm in females), the presence of orange markings on the parotoids (vs absent), and the presence of orange coloration on the dorsolateral glandular warts (vs absent); from *T.notialis* by different color pattern on head and vertebral ridge (orange vs dark brown), lower lip with orange marking (vs brown), and dorsolateral glandular warts and vertebral ridge distinctively large (vs small); from *T.panhai* by having a different ground color (black vs dark brown to brown), the presence of a distinct black line extending from the back of the eye towards the shoulder (vs less evident brownish line to absent line), dorsal edges of tail black (vs yellow, orange to reddish brown). Since *T.panhai* is the closest known taxon to *Tylototritonngoclinhensis* sp. nov., additional morphological comparisons were made between the two species, especially between topotypic *T.panhai* type I and type II from Laos. Tail height was the only character (with n ≥ 3) that showed variation, presenting higher values both in males and in females (Fig. 8) of *T.panhai*, in relation to the new species.

The new species is distinguished from *T.pasmansi* by having rib nodules distinctively large (vs small), the presence of orange markings on the parotoid (vs absent), and the presence of orange colorations of the dorsolateral glandular warts (vs absent); from *T.sini* by different color pattern on head and vertebral ridge (orange vs dark brown), the presence of orange markings on the parotoids (vs absent), the presence of orange coloration on the dorsolateral glandular warts (vs absent), and dorsolateral glandular warts distinct and large (vs small); from *T.sparreboomi* by different color pattern on head and vertebral ridge (orange vs dark brown), the presence of orange markings on the parotoids (vs absent), the presence of orange coloration on the dorsolateral glandular warts (vs absent), and tips of fingers reaching between eye and nostril when foreleg laid forward (vs reaching nostril); from *T.thaiorum* by having head longer than wide (vs head wider than long), the presence of orange markings on the parotoids (vs absent), and the presence of orange coloration on the dorsolateral glandular warts (vs absent); from *T.tongziensis* by different shape of dorsolateral glandular warts (knob-like vs slightly flattened), the presence of orange markings on the parotoids (vs absent), and the presence of orange coloration on the dorsolateral glandular warts (vs absent); from *T.vietnamensis* by different shape of dorsolateral glandular warts (knob-like vs slightly flattened), the presence of orange markings on the parotoids (vs absent), the presence of orange coloration on the dorsolateral glandular warts (vs absent), and the presence of gular fold (vs absent); from *T.wenxianensis* by different shape of dorsolateral glandular warts (knob-like vs slightly flattened), the presence of orange markings on the parotoids (vs absent), the presence of orange coloration on the dorsolateral glandular warts (vs absent), and finger tips reaching to between eyes and nostrils (vs reaching nostril); from *T.ziegleri* by having head longer than wide (vs head wider than long), different color pattern on head and vertebral ridge (orange vs dark brown), the presence of orange markings on the parotoids (vs absent), and the presence of orange coloration on the dorsolateral glandular warts (vs absent); from *T.anguliceps*, *T.himalayanus*, *T.kachinorum*, *T.ngarsuensis*, *T.panwaensis*, *T.phukhaensis*, *T.podichthys*, *T.pulcherrimus*, *T.pseudoverrucosus*, *T.shanorum*, *T.shanjing*, *T.uyenoi*, *T.umphangensis*, and *T.verrucosus* by having limbs and tail edges dark brown except for the orange digits, palms, and soles (vs limbs and tail edges uniformly orange or pale brown in the latter), the presence of a black line extending from the back of the eye towards the shoulder (vs absent); and from *T.kweichowensis* and *T.yangi* by different color pattern on tail (black vs uniformly orange in the latter), and ventral side dark brown (vs ventrolateral sides yellow in the latter).

## ﻿Discussion

New species are being continuously described within the genus *Tylototriton*. A total of 12 new species has been recorded during the last three years alone, from China, Thailand and northern Vietnam (Bernardes et al. 2020; Li et al. 2020; Li et al. 2022; Luo et al. 2022; Lyu et al. 2021; Pomchote et al. 2021; Poyarkov et al. 2021a; Dufresnes and Hernandez 2022; Rao 2022; Wang et al. 2022). Most recent descriptions were due to the separation of species complexes, that were previously masked by phenotypic similarities (Bernardes et al. 2020; Li et al. 2020; Lyu et al. 2021; Pomchote et al. 2021; Poyarkov et al. 2021a; Luo et al. 2022). There certainly is high potential to continue uncovering new species by applying integrative taxonomic analyses (Dufresnes and Hernandez 2022). However, some novel species descriptions occur by conducting field work in previously unexplored areas. In our case, a new species was discovered in a region where several field surveys to assess the herpetological diversity had been conducted in the past (exp. Orlov 2005, 2009; Jenkins et al. 2007; David et al. 2011). However, individuals of the new species have been only found in a recent field survey in 2022.

This is also the first time that a crocodile newt species is recorded from the Central Highlands of Vietnam. Occurring at an elevation more than 1,800 m, this discovery sets an altitudinal record for the genus in the country, with former ranges distributed between 250 m (for *T.vietnamensis*) and 1,740 m (for *T.anguliceps*). Furthermore, this discovery represents the southernmost distribution range of the genus known to date. The new species is located approximately 370 air km distant from the nearest *T.notialis* population from Khammouan Province, Laos. Ngoc Linh Mountain is located on the northwestern border of the Kon Tum Massif and is the highest peak in Central Vietnam with 2,598 m (Sterling et al. 2006). The new species is also expected to be found in other localities on the Kon Tum Plateau. The climate in Ngoc Linh Mountain is relatively cool, as it is part of a tropical mountain system (averages of 15–18.5 °C throughout the year). Humidity is high, with strong amounts of rainfall (ranging from 2,600 to 3,200 mm per year) and distinct cloud coverage. Due to this unique climate and geographical position, Ngoc Linh has become a hotspot of amphibian diversity, with numerous endemic species. The area is also the type locality for some recently discovered species, namely *Leptobrachiumngoclinhense* (Orlov 2005), *Thelodermanebulosum* Rowley, Le, Hoang, Dau & Cao, 2011, *Leptobrachellafirthi* (Rowley, Hoang, Dau, Le & Cao, 2012), *Gracixaluslumarius* Rowley, Le, Dau, Hoang & Cao, 2014, and *G.trieng* Rowley, Le, Hoang, Cao & Dau, 2020 (Orlov 2005; Rowley et al. 2011, 2012, 2014, 2020). Most recently Krzikowski et al. (2022) highlighted the extraordinary endemism rate of amphibians in the Central Highlands of Vietnam, where the highest species diversity, with 130 species, was recorded among the eight regions of Vietnam while also containing the highest number of regionally occurring, micro-endemic amphibians, amounting for 26 species, for example: *Leptobrachellacrocea* (Rowley, Hoang, Le, Dau & Cao, 2010) and *Microhyladarevskii* Poyarkov, Vassilieva, Orlov, Galoyan, Tran, Le, Kretova & Geissler, 2014, next to the afore-mentioned species (Krzikowski et al. 2022; Frost 2023). Thus, the discovery dealt with herein is another and very remarkable case, demonstrating that the Central Highlands play a special role in Vietnamese amphibian diversification and evolution.

Ngoc Linh Nature Reserve has been established in 1986 with an initial protected area of 41,424 hectares and represents a key biodiversity area for the threatened Golden-winged Laughingthrush, *Trochalopteronngoclinhense*, listed as Endangered in the IUCN Red List, as well as for other rare species like the Truong Son Muntjac, *Muntiacustruongsonensis*, Rhesus Macaque, *Macatamulatta*, and the Stump-tailed Macaque, *Macacaarctoides* (Le et al. 1998; BirdLife International 2023). The Ngoc Linh Crocodile Newt certainly will represent another flagship species of this protected area and its surroundings, as individuals have been found both inside and outside of the Ngoc Linh Nature Reserve.

Together with *T.panhai*, *T.ngoclinhensis* is the only known species within the subgenus Yaotriton to present characteristic colorful markings on the body. Although there is a high phenotypic variation recorded for *T.panhai* (types I, II, and III from Hernandez 2016), *T.ngoclinhensis* is clearly distinguishable both morphologically and phylogenetically from the former. Nevertheless, further studies should be conducted to obtain additional data on population characteristics, including further biogeographical analyses.

*Tylototriton* species so far have been documented as having little dispersal abilities due to limitations in vagility and their specific habitat requirements (Zamudio and Wieczorek 2007). Therefore, the discovery of this new species geographically separated by more than 300 km from all known congeners, and bearing a particularly unique colorful pattern represents not only an important discovery in terms of evolution and zoogeography, but also reveals to be of high conservation relevance.

### ﻿Conservation recommendation

Due to the aforementioned reasoning and given that *Tylototritonngoclinhensis* sp. nov. is currently known only from Ngoc Linh Mountain, implying a limited distribution range composed of a single small isolated mountain population, is distinct evidence for a high threat potential. In addition to its special zoogeographic situation and rarity, the particular colorful appearance of the new crocodile newt species is very likely to draw the interest of illegal collectors. Therefore, this species should be provisionally considered to be listed as Endangered (EN) under IUCN Red List criteria B1ab(i,iii), as it is known only from Ngoc Linh Mountain in Kon Tum Province; the estimated extent of occurrence (EOO) is less than 500 km^2^; and the species' habitat is currently being degraded due to human impacts, for example forest product exploitation, tourism development and agricultural cultivation. All species of the genus *Tylototriton* are listed in the Appendices of the Convention on International Trade in Endangered Species of Wild Fauna and Flora (CITES 2022) and also in the Governmental Decree No. 84/2021/ND-CP of Vietnam and therefore the new species is automatically protected under these regulations.

## Supplementary Material

XML Treatment for Tylototriton ngoclinhensis

## ﻿Additional information

### Author ORCIDs

Trung My Phung https://orcid.org/0000-0001-7086-8110

Cuong The Pham https://orcid.org/0000-0001-5158-4526

Truong Quang Nguyen https://orcid.org/0000-0002-6601-0880

Huy Quoc Nguyen https://orcid.org/0000-0003-3171-1561

Marta Bernardes https://orcid.org/0009-0008-2847-8862

Thomas Ziegler https://orcid.org/0000-0002-4797-609X

Tao Thien Nguyen https://orcid.org/0000-0002-5640-4536

### Data availability

All of the data that support the findings of this study are available in the main text or Supplementary Information.
