# ﻿Corrigendum: Phung TM, Pham CT, Nguyen TQ, Ninh HT, Nguyen HQ, Bernardes M, Le ST, Ziegler T, Nguyen TT (2023) ﻿Southbound – the southernmost record of *Tylototriton* (Amphibia, Caudata, Salamandridae) from the Central Highlands of Vietnam represents a new species. ZooKeys 1168: 193-218. https://doi.org/10.3897/zookeys.1168.96091

**DOI:** 10.3897/zookeys.1261.175115

**Published:** 2025-12-02

**Authors:** Trung My Phung, Cuong The Pham, Truong Quang Nguyen, Hoa Thi Ninh, Huy Quoc Nguyen, Marta Bernardes, Son Thanh Le, Thomas Ziegler, Tao Thien Nguyen

**Affiliations:** 1 Dong Khoi 9A, Tam Hiep, Dong Nai Province, Vietnam Unaffiliated Tam Hiep Vietnam; 2 Institute of Biology, Vietnam Academy of Science and Technology, 18 Hoang Quoc Viet Road, Hanoi 10072, Vietnam Institute of Biology, Vietnam Academy of Science and Technology Hanoi Vietnam; 3 Chengdu Institute of Biology, Chinese Academy of Sciences, Chengdu, Sichuan, China Chengdu Institute of Biology, Chinese Academy of Sciences Chengdu China; 4 Cologne Zoo, Riehler Str. 173, D–50735 Cologne, Germany Cologne Zoo Cologne Germany; 5 Institute of Zoology, University of Cologne, Zülpicher Str. 47b, D–50674 Cologne, Germany University of Cologne Cologne Germany; 6 National Institute of Medicinal Materials, 43 Đinh Tien Hoang, Ho Chi Minh City, Vietnam National Institute of Medicinal Materials Ho Chi Minh City Vietnam

This corrigendum addresses an error in Phung et al. (2023), wherein we described *Tylototriton ngoclinhensis*, a new species from Ngoc Linh Mountain, Central Highlands, Vietnam. In the original publication, the GenBank accession numbers for three sequences of the new species were incorrectly listed in Table 1. The incorrect numbers, LC575221–LC575223, should be replaced with the correct numbers, LC757221–LC757223. An updated version of Table 1 is provided herein. The authors sincerely apologize for this oversight. 

**Table 1.** Samples of the *Tylototriton* species and other species used for DNA analysis in this study.

No.	Scientific name	Voucher number	Locality	Genbank no	reference
1.	*Tylototriton ngoclinhensis* sp. nov.	IEBR A.5131	Kon Tum Prov., Vietnam	LC757223	This study
2.	*Tylototriton ngocinhensis* sp. nov.	IEBR A.5130	Kon Tum Prov., Vietnam	LC757221	This study
3.	*Tylototriton ngoclinhensis* sp. nov.	IEBR A.5133	Kon Tum Prov., Vietnam	LC757222	This study
4.	*T. anguliceps*	NUOL 00420	Viengphoukha, Luang Namtha, Laos	KT304301	Phimmachak et al. (2015)
5.	*T. anhuiensis*	CIB 08042905-3	Yuexi Co. Anhui Prov., China	KY800854	Nishikawa et al. (2013)
6.	*T. anhuiensis*	CIB 08042905-4	Yuexi Co. Anhui Prov., China	KY800855	Nishikawa et al. (2013)
7.	*T. asperrimus*	CIB GX20080714	Jinxiu Co., Guangxi Prov., China	KY800819	Nishikawa et al. (2013)
8.	*T. broadoridgus*	CIB 200084	Sangzhi Co., Hunan Prov., China	KY800837	Nishikawa et al. (2013)
9.	*T. dabienicus*	HNNU1004-024	Shangcheng Co., Henan Prov., China	KC147812	Nishikawa et al. (2014)
10.	*T. dabienicus*	HNNU 1004-015	Shangcheng Co., Henan Prov., China	KC147811	Nishikawa et al. (2014)
11.	*T. dabienicus*	HNNU 1004-026	Shangcheng Co., Henan Prov., China	KY800869	Nishikawa et al. (2013)
12.	*T. daloushanensis*	GZNU 20060626002	Suiyang Co., Guizhou Prov., China	JF825872	Li et al. (2022)
13.	*T. daloushanensis*	GZNU 20060626001	Suiyang Co., Guizhou Prov., China	FJ415600	Wang and Gu (2008)
14.	*T. hainanensis*	CIB 20081048	Mt. Diaoluo, Hainan Prov., China	KC147817	Nishikawa et al. (2013)
15.	*T. himalayanus*	CIB 201406246	Mai Pokhari, Illam, Mechi, Nepal	KT765173	Khatiwada et al. (2015)
16.	*T. kweichowensis*	CIB Wg20080818014	Bijie City, Guizhou Prov., China	KY800823	Nishikawa et al. (2015)
17.	*T. liuyangensis*	CIB 110601F06	Liuyang City, Hunan Prov., China	KY800875	Yang et al. (2014)
18.	*T. maolanensis*	CIB ML20180427003	Libo Co., Guizhou Prov., China	MK820701	Li et al. (2020)
19.	*T. maolanensis*	CIB ML20180427004	Libo Co., Guizhou Prov., China	MK820702	Li et al. (2020)
20.	*T. maolanensis*	GZNU 200706050101	Leishan Co., Guizhou Prov., China /	FJ415596	Li et al. (2020)
21.	*T. maolanensis*	GZNU 200706050102	Leishan Co., Guizhou Prov., China /	JF825868	Li et al. (2020)
22.	*T. ngarsuensis*	LSUHC13762	Shan State, Myanmar	MH836585	Grismer et al. (2018)
23.	*T. notialis*	FMNH: HERP:271120	Boualapha, Khammouan, Laos	HM462061	Poyarkov et al. (2021)
24.	*T. panhai*	NUOL 00424	Botene, Xaignabouli, Laos	KT304309	Phimmachak et al. (2015)
25.	*T. panhai*	NUOL 00425	Botene, Xaignabouli, Laos	KT304311	Phimmachak et al. (2015)
26.	*T. panhai*	NUOL 00421	Botene, Xaignabouli, Laos	KT304310	Phimmachak et al. (2015)
27.	*T. pasmansi*	IEBR 4466 (Holotype)	Da Bac, Hoa Binh Prov., Vietnam	MT210166	Bernardes et al. (2020)
28.	*T. pasmansi*	IEBR:4467	Da Bac, Hoa Binh Prov., Vietnam	MT210167	Bernardes et al. (2020)
29.	*T. podichthys*	NCSM 77725	Phoukhoun, Luang Phabang, Laos	KT304295	Phimmachak et al. (2015)
30.	*T. pseudoverrucosus*	CIB WCG2012003	Ningnan Co., Liangshanyizu state, Sichuan Prov., China	KY800861	Nishikawa et al. (2015)
31.	*T. pulcherrimus*	KUHE:46406	Pet Trade	KY800880	Nishikawa et al. (2013)
32.	*T. shanjing*	CIB 980004	Baoshan City, Yunnan Prov., China	KY800831	Nishikawa et al. (2013)
33.	*T. shanorum*	KUHE 42348	Shan State, Myanmar	AB769544	Bernardes et al. (2017)
34.	*T. sini*	SYS a008354	Mt Yunkai, Guangdong Prov., China	OK539836	Lyu et al. (2021)
35.	*T. sparreboomi*	IEBR 4476 (Holotype)	Sin Ho, Lai Chau Prov., Vietnam	MT210162	Bernardes et al. (2020)
36.	*T. taliangensis*	CIB GG200110183	Shimian Co., Yan’an City, Sichuan Prov., China	KC147819	Nishikawa et al. (2015)
37.	*T. thaiorum*	ZMMU A-7577	Pu Hoat NR, Nghe An Prov., Vietnam	MW883478	Polyakov et al. (2021)
38.	*T. tongziensis*	CIB WB2020081511	Tongzi Co., Guizhou Prov., China	OK349411	Li et al. (2022)
39.	*T. tongziensis*	CIB TZ20160714002	Tongzi Co., Guizhou Prov., China	OK349413	Li et al. (2022)
40.	*T. tongziensis*	TZ20160714010	Tongzi Co., Guizhou Prov., China	OK349414	Li et al. (2022)
41.	*T. tongziensis*	CIB WB2020202	Tongzi Co., Guizhou Prov., China	OK349415	Li et al. (2022)
42.	*T. uyenoi*	KUHE:19147	Doi Suthep, Chiang Mai Prov., Thailand	AB830733	Nishikawa et al. 2013
43.	*T. verrucosus*	CIB-TSHS1	Longchuan Co., Dehong state, Yunnan Prov., China	KY800847	Nishikawa et al. 2013
44.	*T. wenxianensis*	CIB 2010123101	Pingwu Co., Gansu Prov., China	KY800867	Nishikawa et al. 2017
45.	*T. wenxianensis*	CIB 2010123102	Pingwu Co., Gansu Prov., China	KY800868	Nishikawa et al. 2017
46.	*T. yangi*	KUHE:42282	Pet Trade	KY800887	Nishikawa et al. 2014
47.	*T. ziegleri*	VNMN 3390	Quan Ba, Ha Giang Prov., Vietnam	KY800889	Nishikawa et al. 2013
48.	*T. liuyangensis*	CSUFT20100108	Liuyang City, Hunan Prov., China	KJ205598	Yang et al. 2014
49.	*T. phukhaensis*	CUMZ-A-7717	DPKNP, Nan Prov., Thailand	MN912573	Pomchote et al. 2020
50.	*T. sparreboomi*	IEBR 4477	Sin Ho, Lai Chau Prov., Vietnam	MT210163	Bernardes et al. (2020)
51.	*T. vietnamensis*	IEBR A.2014.44	Taunggyi Township, Shan State, Myanmar	KX609962	Bernardes et al. 2017
52.	*T. vietnamensis*	IEBR A.2014.43	Hoanh Bo, Quang Ninh Prov., VN	KX609961	Bernardes et al. 2017
53.	*T. vietnamensis*	IEBRA.0701	Mau Son, Lang Son Prov., Vietnam	KY800873	Bernardes et al. 2017
54.	*Echinotriton chinhaiensis*	CIB ZHJY2	Zhenhai Co., Zhejiang Prov., China	KY800892	Nishikawa et al. (2014)
55.	*Pleurodeles waltl*	-	Cadiz, Andalusia, Spain	EU880330	Zhang et al. (2008)

## ﻿Additional information

### Use of AI

No use of AI was reported.

### Author ORCIDs

Trung My Phung https://orcid.org/0000-0001-7086-8110

Cuong The Pham https://orcid.org/0000-0001-5158-4526

Truong Quang Nguyen https://orcid.org/0000-0002-6601-0880

Huy Quoc Nguyen https://orcid.org/0000-0003-3171-1561

Marta Bernardes https://orcid.org/0009-0008-2847-8862

Thomas Ziegler https://orcid.org/0000-0002-4797-609X

Tao Thien Nguyen https://orcid.org/0000-0002-5640-4536

### Data availability

All of the data that support the findings of this study are available in the main text.
